# Cancer bronchique à petites cellules et grossesse: à propos d'un cas avec revue de la literature

**DOI:** 10.11604/pamj.2016.23.130.7856

**Published:** 2016-03-25

**Authors:** Fatima Safini, Hassan Jjouhadi, Asmaa Chehal, Farida Mernissi, Akpoo Wilfried, Zineb Bouchbika, Amina Taleb, Nadia Benchakroun, Nezha Tawfiq, Souha Sahraoui, Abdellatif Benider

**Affiliations:** 1 Service de Radiothérapie-Oncologie, Centre Hospitalier Universitaire Ibn Rochd, Casablanca, Maroc; 2 Service d'Anatomie et de Cytologie Pathologiques, Centre Hospitalier Universitaire Ibn Rochd, Casablanca, Maroc

**Keywords:** Cancer bronchique, cancer bronchique à petites cellules, grossesse, Bronchial carcinoma, small cell bronchial carcinoma, pregnancy

## Abstract

Le cancer broncho-pulmonaire (CBP) de la femme enceinte est une entité rare, d’évolution péjorative. Cette situation devient de plus en plus fréquente, du fait de l'augmentation du tabagisme chez la femme. La transmission tumorale trans-placentaire avec atteinte fœtale est décrite surtout chez les femmes non traitées. Le traitement est multidisciplinaire et n'est pas bien codifié. Nous rapportons le cas d'une patiente de 23 ans chez qui le diagnostic d'un carcinome bronchique à petites cellules a été fait au cours de sa grossesse. Elle avait bénéficié d'une chimiothérapie pendant la grossesse, bien tolérée. L’évaluation radiologique a objectivé une stabilisation du processus pulmonaire. Le traitement a été complété par une association radio-chimiothérapie concomitante après l'accouchement.

## Abstract (en)

Bronchopulmonary cancer (CBP) in pregnant woman is a rare entity with a poor outcome. This situation is becoming increasingly common, due to the increase in smoking among women. Transplacental transmission of tumor associated with fetal damage is described especially among untreated women. Treatment is multidisciplinary and it is not well codified. We report the case of a 23 year old patient with small cell bronchial carcinoma diagnosed during pregnancy. She had received chemotherapy during pregnancy and it was well tolerated. Radiological evaluation objectified a stabilization of the pulmonary process. The treatment was completed with concomitant radiochemotherapy after delivery.

## Introduction

L'association CBP et grossesse est extrêmement rare et représente 11000 à 11500. Elle devient de plus en plus fréquente, du fait de l'augmentation du tabagisme chez la femme [1]. Nous rapportons le cas d'une patiente enceinte présentant un carcinome bronchique à petites cellules.

## Patient et observation

**Figure 1:**
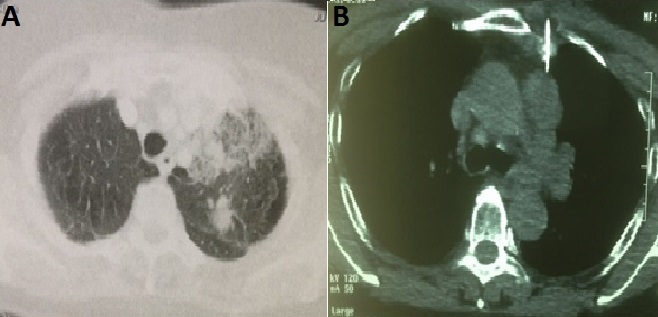
A) Scanner thoracique fenêtre parenchymateuse objectivant un processus médiastino-pulmonaire supérieur avec présence d'un nodule satellite contours spiculées; B) coupe scannographique fenêtre médiastinale.

**Figure 2:**
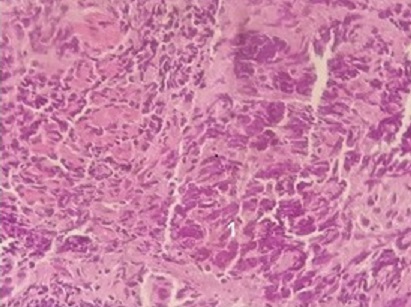
Coupe histologique montrant l'aspect d'un infiltrat carcinomateux peu différencié à petites cellules (1), coloration HE×10

**Figure 3:**
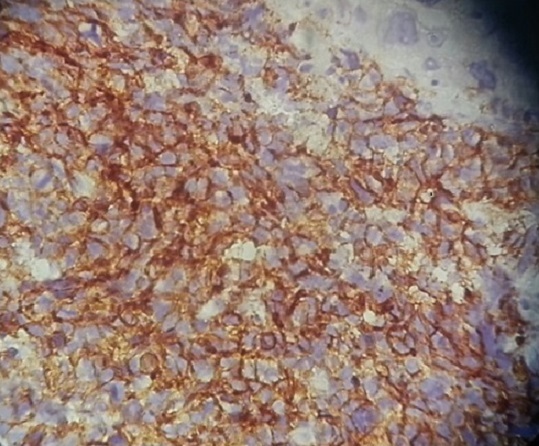
Positivité de l'immuno-marquage au CD56 d'expression membranaire, grossissement×40

**Figure 4:**
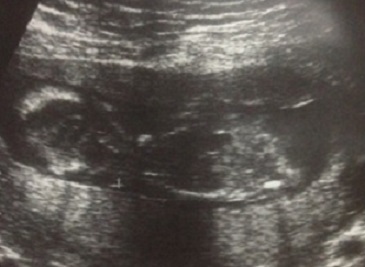
Echographie obstétricale objectivant une grossesse mono-fœtale évolutive de 10 SA

Patiente âgée de 23 ans, mariée, nullipare, sans antécédents pathologiques notable et sans notion de tabagisme actif ni passif, ayant travaillé dans une usine de confection pendant 10 ans. Elle avait consulté au service de pneumologie pour des douleurs de l'hémithorax gauche associées à une dyspnée, sans hémoptysie. Le tout évoluant dans un contexte d'altération de l’état général avec amaigrissement non chiffré. L'examen clinique trouvait une patiente dyspnéique en mauvais état général. La TDM thoracique avait confirmé la présence d'un processus médiastino-pulmonaire supérieur gauche inhomogène de 65×38mm arrivant au contact du bord gauche du médiastin et étendu jusqu'au niveau hilaire, avec présence d'un nodule satellite de 22mm de contours spiculées et d'une adénopathie hilaire homolatérale de 15mm (Figure 1(A); Figure 1(B)). La fibroscopie bronchique avait montré un aspect normal de tout l'arbre bronchique sans bourgeon individualisable. Les biopsies bronchiques ainsi que l'aspiration bronchique étaient négatives. Une biopsie transpariétale scannoo-guidée avait confirmé le diagnostic d'une prolifération tumorale maligne peu différenciée à petites cellules (Figure 2). L’étude immunohistochimique était compatible en premier avec un carcinome endocrine à petites cellules devant la positivité de la synaptophysine et du CD56 (Figure 3), la négativité du CD99, de la desmine et de PS 100. Au cours de son hospitalisation, la patiente avait mentionné un retard de règle ayant motivé une échographie pelvienne qui avait confirmé une grossesse monofoetale évolutive de 10 semaines d'aménorrhés (SA) (Figure 4). Le bilan d'extension comportant une IRM cérébrale et une échographie abdominale était normal. Une interruption thérapeutique de grossesse a été proposée, mais refusée par la patiente. Une chimiothérapie systémique a été démarrée à 14 SA, associant adriamycine et cyclophosphamide tous les 21 jours. La patiente a reçu trois cycles de chimiothérapie pendant la grossesse. L’évaluation radiologique par une IRM thoracique avait objectivé une stabilisation du processus pulmonaire. L'accouchement a eu lieu à 37SA par voie haute (césarienne), et a donné naissance à un bébé de sexe féminin, Apgar 10/10, en bon état général. L’étude anatomopathologique du placenta était normale. La patiente a reçu deux mois après l'accouchement une radio-chimiothérapie concomitante à une dose de 60 Gy. L’évolution a été marquée par le décès de la patiente 6 mois après la fin du traitement suite à une progression locale et métastatique.

## Discussion

Le cancer bronchique de la femme enceinte est une entité rare, souvent rapportée sous forme de cas cliniques, ce qui sous-estime son incidence [2]. Le risque de survenue d'un cancer bronchique chez une femme enceinte varie entre 0,07% et 0,1%, soit environ 1 pour 1000 à 1500 grossesse. Le type histologique le plus rapporté dans la littérature en association avec la grossesse est le carcinome bronchique non à petites cellules avec une fréquence de 77%. Le cancer bronchique à petites cellules est moins fréquent et le plus souvent agressif et d'emblée métastatique [1, 3]. Le principal facteur de risque de survenue d'un CBP est le tabagisme. En occident, 30% des femmes en âge de procréer sont fumeuses et parmi elles, 20 à 30% continuent de fumer pendant leur grossesse. Les femmes ont plus de risque de développer un CBP que les hommes. Cela pourrait être expliqué par une sensibilité supérieure aux carcinogènes du tabac chez les femmes, du fait d'un métabolisme différent [3]. Cependant, le tabagisme ne semble pas être le seul facteur expliquant l'augmentation de l'incidence du CBP chez les femmes. D'autres facteurs seront impliqués notamment environnementaux, génétiques telles les mutations activatrices de l'Epidermal Growth Factor Receptor (EGFR) qui sont plus fréquentes chez les femmes non fumeuses. L'influence hormonale est aussi incriminée dans l'histoire naturelle de la maladie. Les œstrogènes ont un effet procarcinogène, alors que la progestérone induit une inhibition de la prolifération cellulaire [1, 3]. En 2005, des données précliniques mettaient en évidence un effet antitumoral de la progestérone. Dernièrement, d'autres recherches prêtent au contraire un rôle de promotion tumorale à la progestérone [4]. Cependant, la plupart des études affirment que les hormones maternelles seraient impliquées dans la rapidité de l’évolution tumorale au cours de la grossesse. La transmission placentaire et/ou f'tale est un phénomène rare. Durant cette dernière décade, 100 cas de métastases placentaires et/ou fœtales ont été rapportés-tous types de cancers confondus. Les cancers les plus en cause étaient: mélanomes (32%), leucémies et lymphomes (15%), cancer du sein (14%) et cancer du poumon (11%) [2, 5]. Malheureusement, cette atteinte n'a pas été recherchée systématiquement dans tous les travaux publiés, alors qu'elle est importante vu sa valeur pronostique et le risque de transmission f'tale [2, 5, 6]. Cette transmission chez le f'tus a été rapportée dans trois cas dont deux étaient secondaires à un cancer à petites cellules de la mère et un seul cas clinique rapporte une greffe métastatique d'un adénocarcinome bronchique au niveau du cuir chevelu du nouveau-né [2, 6]. A noter que ces cas de métastases transmises aux bébés n'ont été retrouvés que chez les patientes non traitées [5]. Les mécanismes incriminés dans la survenue d'une greffe métastatique au niveau du placenta, ou ceux qui seraient susceptibles de l'empêcher, restent hypothétiques. La valeur pronostique d'une métastase placentaire sur l’évolution de la maladie, de la grossesse et le devenir de l'enfant est inconnue. Les connaissances actuelles reposent seulement sur des cas cliniques [7].

Le traitement du cancer du poumon au cours de la grossesse n'est pas codifié comme pour celui par exemple du sein où il existe des recommandations internationales. Il n'y a pas d’étude chez la femme enceinte concernant le traitement optimal [8]. La décision d'une interruption de grossesse doit prendre en considération le stade de la maladie, la probabilité de rémission complète, les drogues qui vont être utilisées et le désir des parents. Certaines femmes enceintes préfèrent reporter le traitement jusqu’à l′accouchement afin de protéger leurs bébés des effets indésirables possibles de la chimiothérapie. Ces femmes devraient être bien informées du risque évolutif de la maladie et ainsi elles peuvent choisir soit l'interruption de grossesse ou une chimiothérapie néoadjuvante qui est sûre après le premier trimestre [3, 9]. D'après les recommandations de l'ESMO, la chimiothérapie peut être administrée aux deuxième et troisième trimestres avec néanmoins un risque majoré de morts intra-utérines, de retard de croissance intra-utérin, de prématurité, d'anomalies mineures du système nerveux central et des gonades [10]. Le Cisplatine et la carboplatine peuvent être utilisés pendant la grossesse avec une efficacité similaire, cependant, une étude récente a montré que le cisplatine peut engendrer quelques effets indésirables chez le fœtus contrairement à la carboplatine [11]. Pour les autres drogues qu'on pourrait associer aux sels de platines, comme etoposide, vinorelbine, paclitaxel, docetaxel, gemcitabine et pemetrexed, peu d’études ont prouvé leur faisabilité au cours de la grossesse [3]. Si la décision de démarrer la chimiothérapie a été prise, il faudrait respecter un délai de trois semaines entre la fin de la dernière cure et l'accouchement, et ne pas administrer de chimiothérapie après la 35ème SA, du fait du risque de myélosuppression entraînant un risque infectieux et hémorragique au moment de l'accouchement [3]. Quant à la radiothérapie pendant la grossesse après le premier trimestre, elle est possible selon des études réalisées sur les lymphomes de Hodgkin chez la femme enceinte [12]. Cependant, dans le cancer du poumon, la majorité des auteurs et l'ESMO recommandent de différer la réalisation de la radiothérapie après l'accouchement. Pour les carcinomes à petites cellules, la médiane de survie est de deux à quatre mois et la survie à cinq ans est de 5-10%. Même si l’évolution clinique très rapide laisse penser que le cancer bronchique est plus grave pendant la grossesse, il n'y a pas de preuve que la grossesse aggrave le pronostic [13].

## Conclusion

Le CBP pendant la grossesse est une pathologie très rare, mais qui sera probablement plus fréquente dans les années à venir vu l’âge tardif des grossesses, l'augmentation de la consommation de tabac et son début précoce chez les jeunes filles [4]. La prise en charge doit être faite par une équipe d'experts incluant gynéco-obstétricien, oncologue, radiothérapeute, chirurgien thoracique, et pédiatre. La place de la chimiothérapie pendant la grossesse reste une option thérapeutique possible. La seule prévention reste l'information et la lutte contre le tabac.
